# Supplementary material for: PLK1 has tumor-suppressive potential in APC-truncated colon cancer cells
Source: Nat Commun. 2018 Mar 16;9:1106. doi: 10.1038/s41467-018-03494-4 (PMC5856809; doi:10.1038/s41467-018-03494-4)
Supplement: Supplementary file 1 — Supplementary Information(PDF 2770 kb) [file 41467_2018_3494_MOESM1_ESM.pdf]

# Supplementary Information:

# PLK1 has tumor-suppressive potential in APC-truncated colon cancer cells

Raab et al.

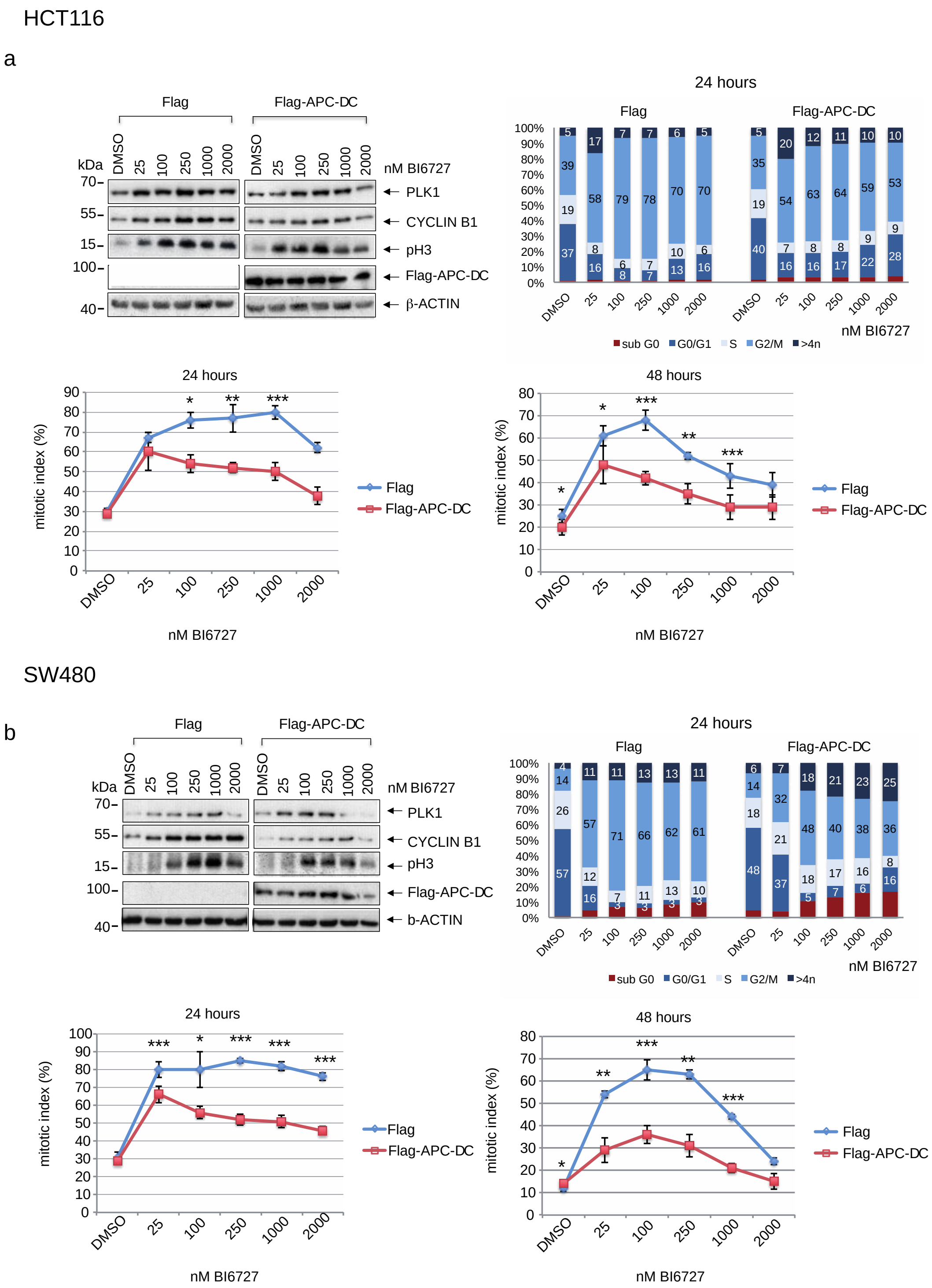


**Supplementary Figure 1: Correlation of PLK1 inhibitor concentrations and mitotic arrest in APC-ΔC-expressing colon cells.**

(Upper left) Lysates of control and APC-ΔC-expressing cells **(a)** HCT116 and **(b)** SW480 treated with BI6727 at increasing concentrations (25–2000 nM) followed by immunoblotting for PLK1, Cyclin B1, phosho-Histone H3 (pH3), Flag-APC-ΔC and β-Actin. (Upper right) The representative quantification of the cell cycle analysis by FACS is shown. (Lower) The mitotic indices of cells treated with increasing concentrations of BI6727 for 24 h and 48 h is depicted. (means±s.d., *n*=3, for each concentration). **P*<0.05, ***P*<0.01, ****P*<0.001, Student's *t*-test, unpaired and two-tailed.

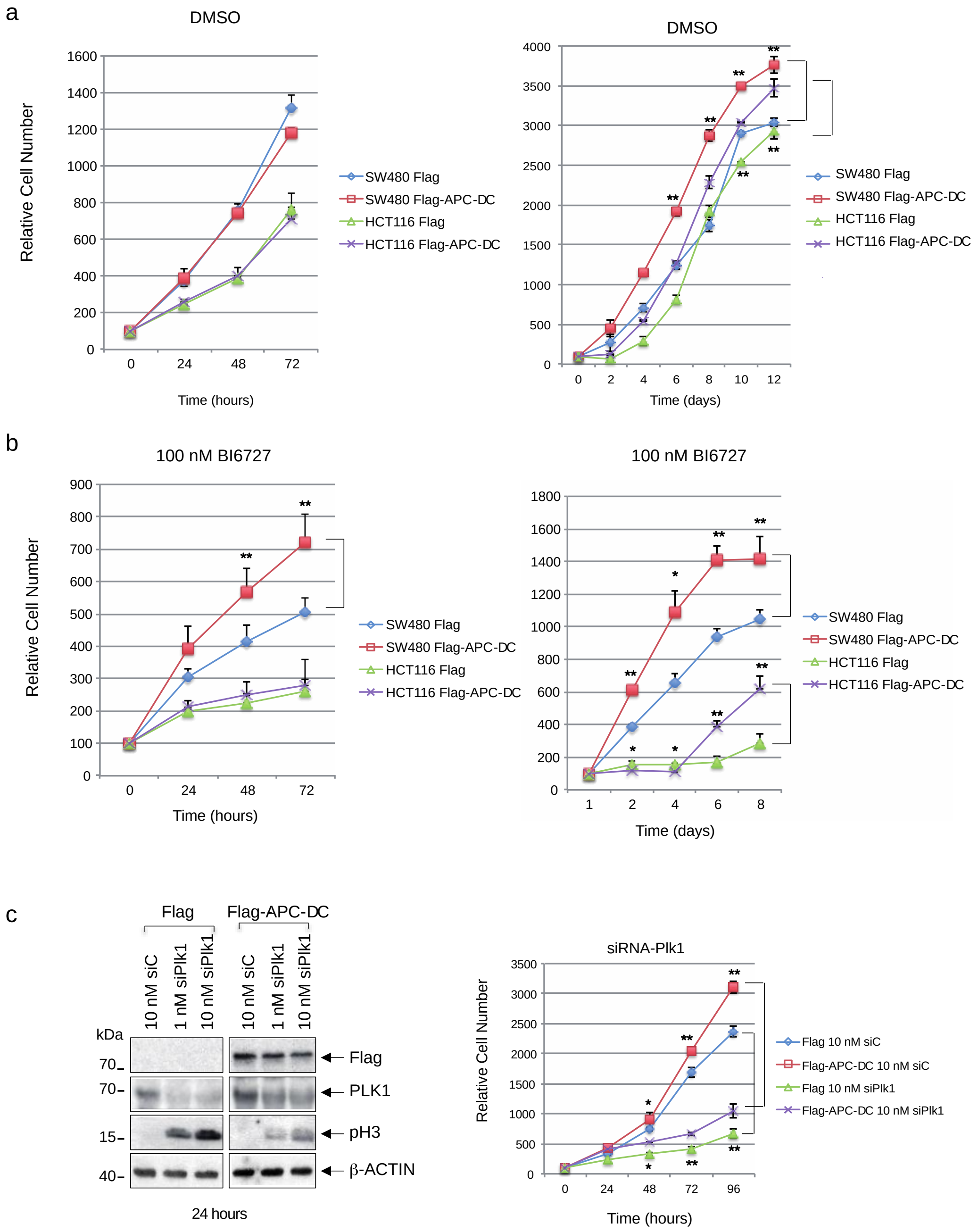


**Supplementary Figure 2: Cellular proliferation of APC-ΔC-expressing, BI6727-treated cells.**

(a) The proliferation of APC-ΔC-expressing HCT116 and SW480 cells compared to control cells over 72 h and 12 d, respectively, was analyzed using an MTT assay. (b) Proliferative activity of APC-ΔC-expressing HCT116 and SW480 cells treated with BI6727 over periods of 72 h and 8 d, respectively (means±s.d., *n*=3, for each concentration). **P*<0.05, ***P*<0.01, Student's *t*-test, unpaired and two-tailed. (c) (Left) Lysates of control and APC-ΔC-expressing SW480 cells transfected with a control siRNA (siC), PLK1-specific siRNA (siPLK1) or treated with BI6727 at 100 nM followed by immunoblotting for Flag-APC-ΔC, PLK1, phosho-Histone H3 (pH3), and β-Actin. (Right) Proliferative activity (over periods of 96 h) of APC-ΔC-expressing SW480 transfected with control siRNA (siC) of siPLK1 (means±s.d., *n*=3, for each concentration). **P*<0.05, ***P*<0.01, Student's *t*-test, unpaired and two-tailed.

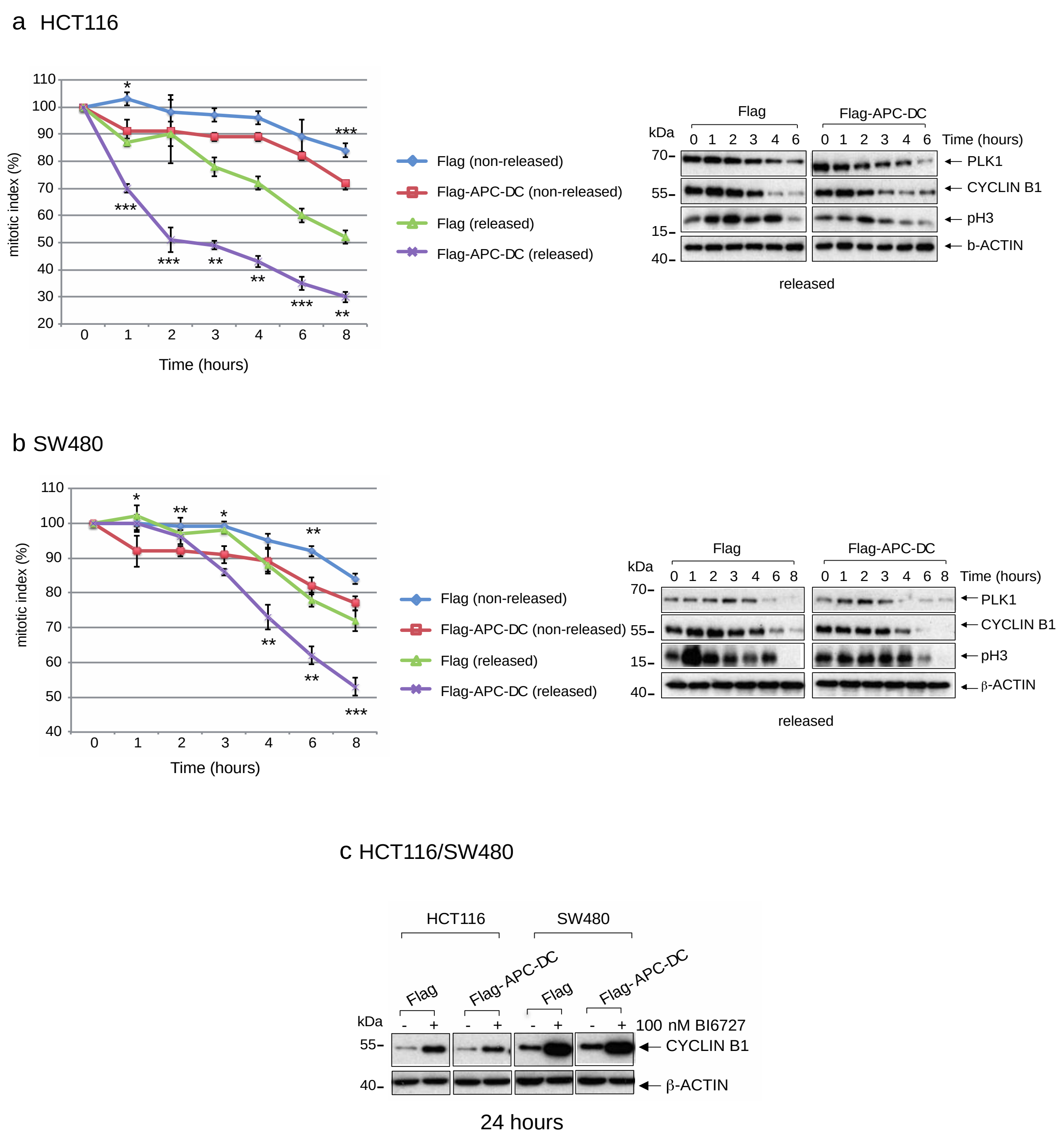


**Supplementary Figure 3: Exit from BI6727-induced mitotic arrest in APC-ΔC-expressing colon cells.**

(Left) Mitotic **(a)** HCT116 and **(b)** SW480 cells with or without APC-ΔC-expression were isolated by shake-off after BI6727 (100 nM)-treatment for 18 h, replated into medium containing 100 nM BI6727 (non-released) or into medium without BI6727 (released) and the mitotic indices were determined (means±s.d., *n*=3, for each concentration). **P*<0.05, ***P*<0.01, ****P*<0.001, Student's *t*-test, unpaired and two-tailed. (Right) Lysates of released cells were immunoblotted for PLK1, Cyclin B1, pH3 and β-Actin. **(c)** Lysates of cells treated with or without 100 nM BI6727 were immunoblotted for Cyclin B1 and β-Actin.

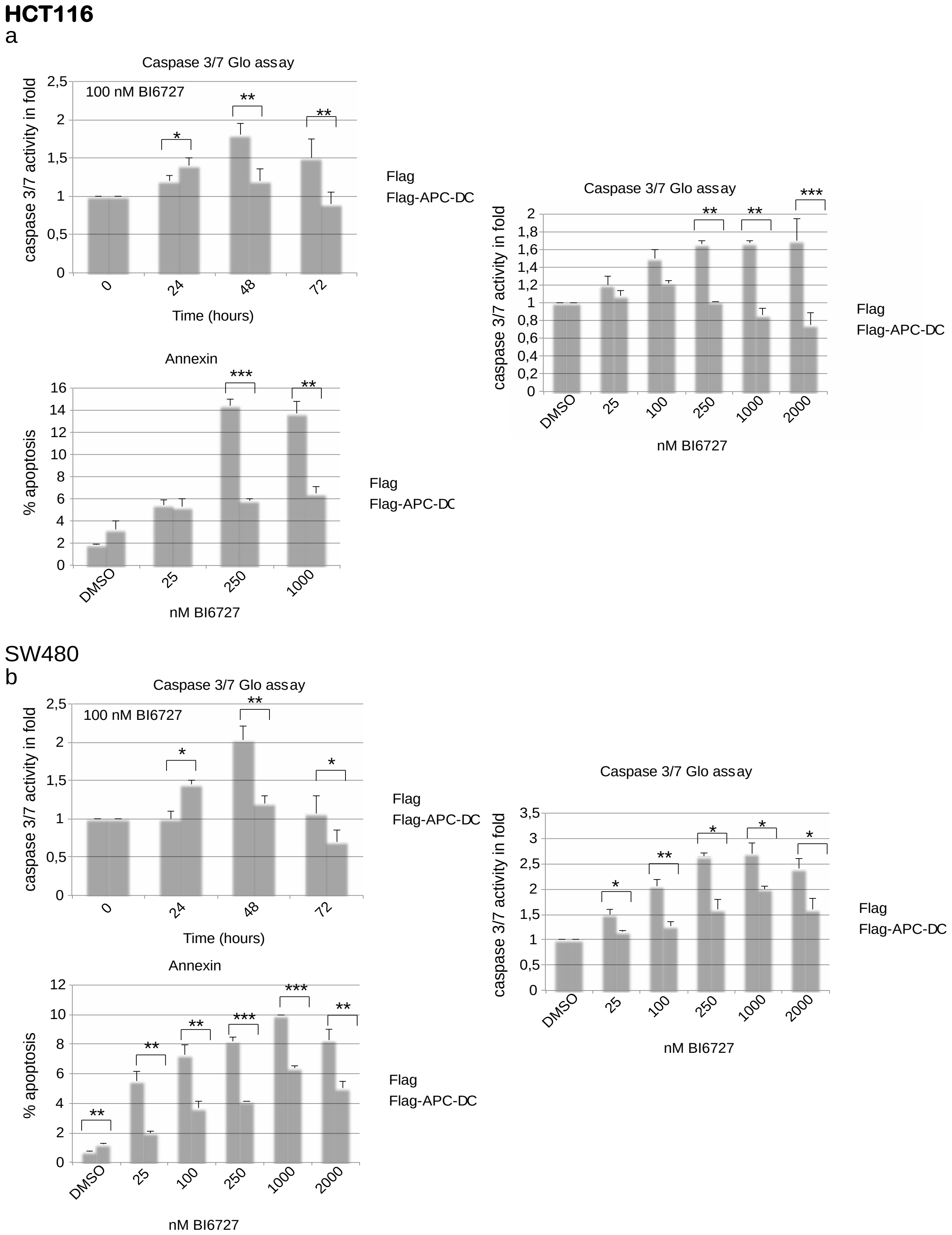


**Supplementary Figure 4: Assessment of apoptosis in APC-ΔC-expressing, BI6727-treated cells.**

APC-ΔC-expressing colon cancer cell lines **(a)** HCT116 and **(b)** SW480 and their control cells were analyzed for apoptotic activity. (Upper left) Caspase-3/7 activity was determined over a period of 72 h in the cell lysates using the Caspase-Glo 3/7 Assay. (Right) Caspase-3/7 activity was determined in the lysates of cells treated with BI6727 at increasing concentrations (25-2000 nM) using the Caspase-Glo 3/7 Assay. (Lower) Annexin V-based measurements were performed using lysates cells treated for 24 h with BI6727 (25-1000 nM) (means±s.d., $n$=3, for each concentration). $^{**}P<0.01$, Student's $t$-test, unpaired and two-tailed.

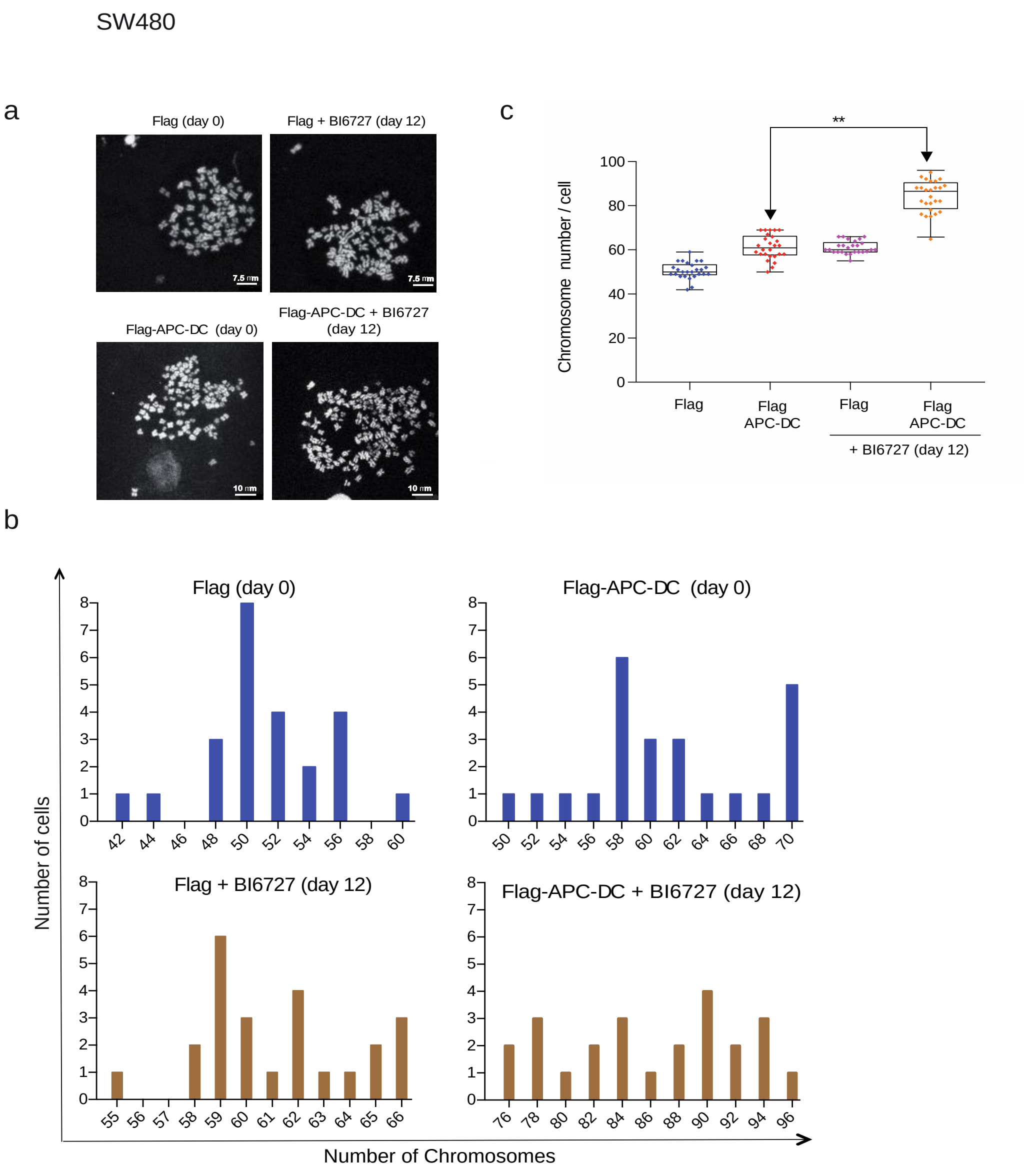


Supplementary Figure 5: **Analysis of chromosomal aberrations in BI6727-treated, APC-ΔC-expressing SW480 cells.**

**(a)** Following the incubation with BI6727 for 48 h and release into fresh medium for 12 d, metaphase spreads of SW480 cells with or without APC-ΔC-expression were prepared and the chromosomes were stained with Hoechst. Scale bar: 7.5 µm and 10 µm **(b)** The histogram plotting of the distribution of chromosome numbers at day 0 and day 12 is shown. **(c)** Quantification of the number of chromosomes in 500 SW480 cells with or without APC-ΔC-expression incubated in the presence or absence of BI6727 (100 nM) for 48 h compared to control cells. (means±s.d., *n*=3, for each test). *$P$<0.05, **$P$<0.01, ***$P$<0.001, Student's *t*-test, unpaired and two-tailed.

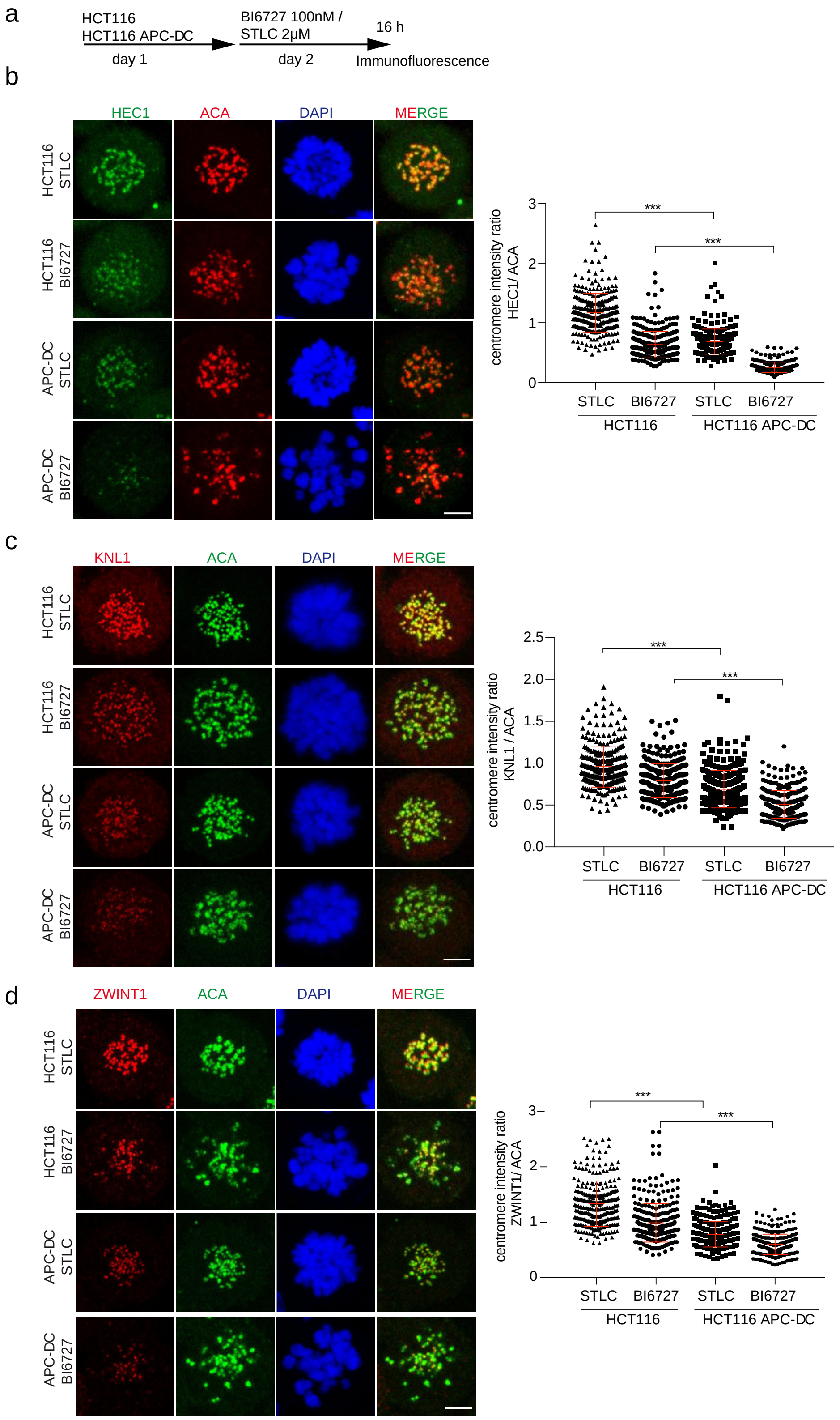


**Supplementary Figure 6: The expression of APC-ΔC reduces the recruitment of HEC1, KNL1 and ZWINT1 to kinetochores.**

(**a**) Scheme of the experimental procedure. APC-ΔC-expressing and control HCT116 cells were seeded on day 1, treated on day 2 either with 2 μM STLC or 100 nM BI6727 for 16 h and harvested on day 3. Cells were fixed, processed for immunofluorescence using antibodies for **(b)** HEC1, **(c)** KNL1, **(d)** ZWINT1 and ACA. Intensities were normalized to ACA and quantified. Scale bar, 5 μm. The intensities were normalized to ACA. Values were calculated from at least 50 cells per treatment and represented as mean±sd. ****P*<0.001, (*n*=3, for each treatment).

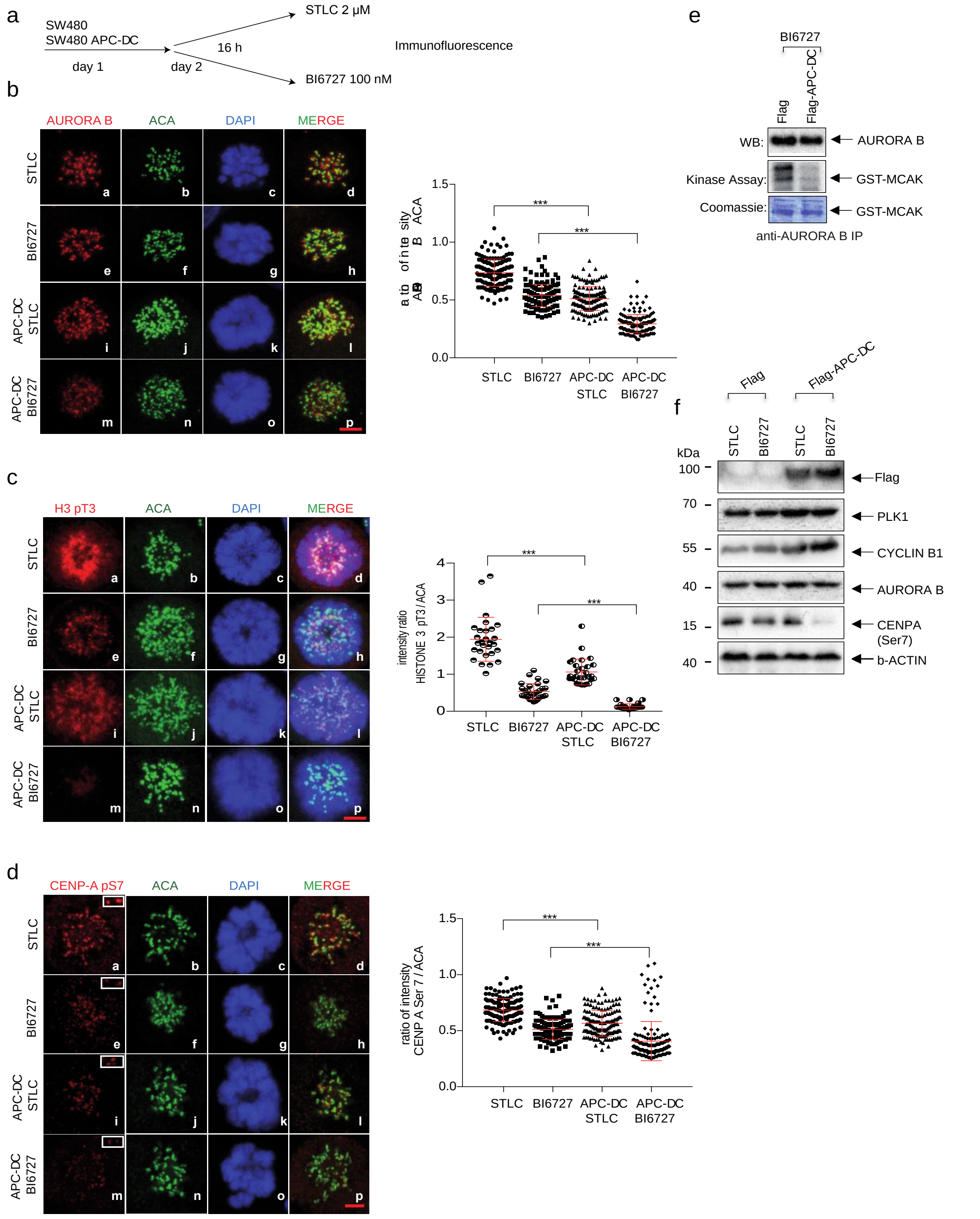


**Supplementary Figure 7**: **The inhibition of PLK1 decreases the levels of Aurora B, CENP-A pS7 and Histone 3 pT3 in cells expressing APC-ΔC.**

**(a)** Scheme of the experimental procedure. APC-ΔC-expressing SW480 and control cells were seeded on day 1, treated on day 2 either with 2 µM STLC or 100 nM BI6727 for 16 h and harvested on day 3. Cells were fixed, processed for immunofluorescence using antibodies for **(b)** Aurora B, **(c)** Histone 3 pT3, **(d)** CENP-A pS7 and ACA antibodies and intensities were normalized to ACA and quantified. Scale bar, 5µm. Values were calculated from at least 50 cells per treatment (means±s.d., $n$=3, for each test). ***$P$<0.001, Student's $t$-test, unpaired and two-tailed. **(e)** The kinase activity of immunoprecipitated Aurora B protein from APC-ΔC-expressing SW480 and control cells treated for 24 h with 100 nM BI6727 using MCAK as substrate was determined. **(f)** Lysates of APC-ΔC-expressing SW480 and control cells treated with STLC or BI6727 were immunoblotted for Flag, PLK1, Cyclin B1, Aurora B, CENPA (Ser7), and β-Actin.

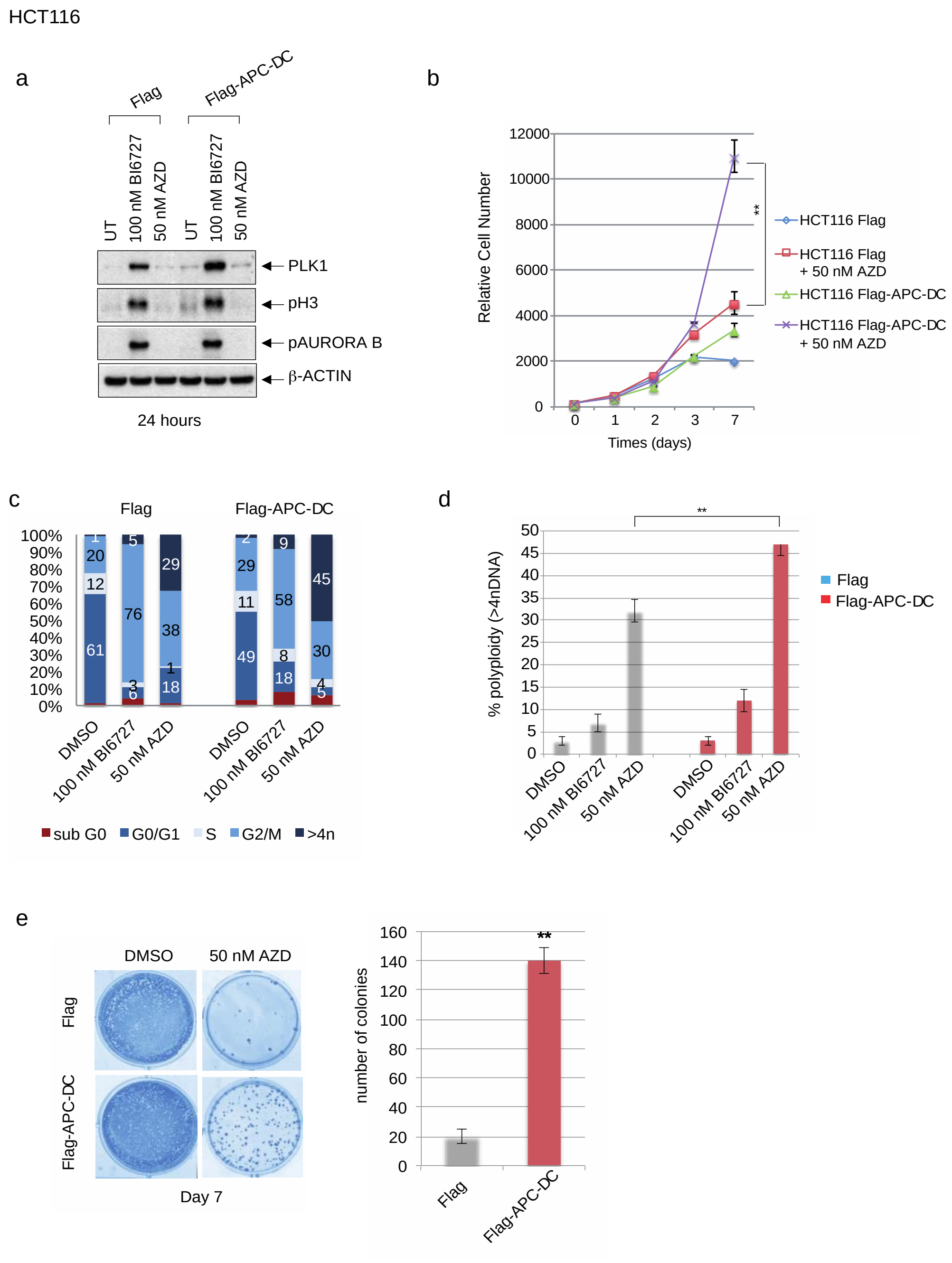


**Supplementary Figure 8: Aurora B inhibition correlated with proliferation, DNA content and colony formation in APC-ΔC-expressing colon cells.**

**(a)** Protein extracts from transfected HCT116 cells were treated with 100 nM BI6727 or 50 nM AZD followed by immunoblotting for PLK1, phosho-Histone H3 (pH3), phospho-Aurora B, and β-Actin. **(b)** The proliferation of APC-ΔC-expressing HCT116 and control cells over 7 d was analyzed using an MTT assay. (means±s.d., *n*=3, for each concentration). **P*<0.05, ***P*<0.01, ****P*<0.001, Student's *t*-test, unpaired and two-tailed. **(c)** The representative quantification of the cell cycle analysis by FACS showing control (DMSO) and APC-ΔC-expressing cells treated for 24 h with 100 nM BI6727 or 50 nM AZD, respectively. **(d)** HCT116 cells with or without APC-ΔC-expression were treated for 48 h with 100 nM BI6727 or 50 nM AZD, respectively and were subsequently subjected to DAPI staining and examination by FACS for the analysis of the DNA content. (means±s.d., *n*=3, for each time point). **P*<0.05, ***P*<0.01, ****P*<0.001, Student's *t*-test, unpaired and two-tailed. **(e)** HCT116 cells with or without APC-ΔC-expression were incubated with AZD (50 nM) for 48 h. On day 2 AZD was washed away, cells were replated in fresh medium, cultivated for 8 days, stained with Coomassie Blue and the number of colonies was determined.

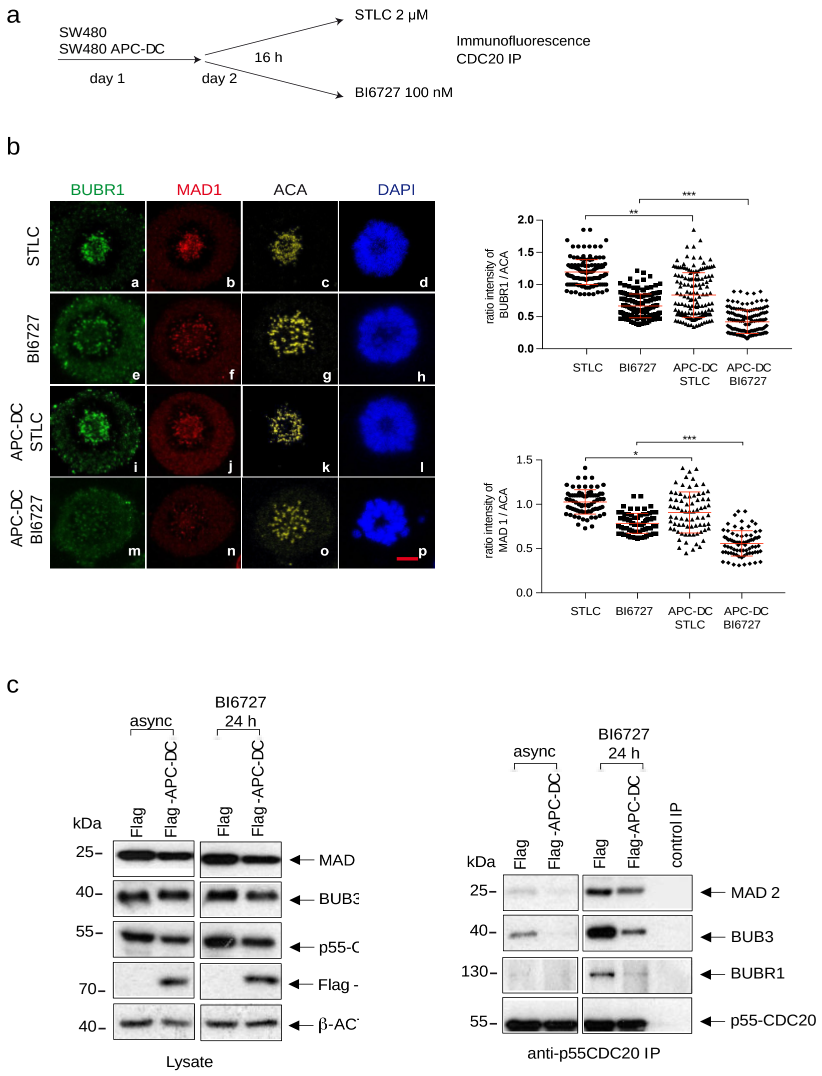


**Supplementary Figure 9**: **Inhibition of PLK1 in APC-ΔC-expressing SW480 cells reduces the recruitment of BUBR1, MAD1 to kinetochore and decreases the mitotic checkpoint complex association.**

**(a)** Scheme of the experimental procedure. **(b)** The kinetochore recruitment of the checkpoint proteins BUBR1 and MAD1 in APC-ΔC-expressing SW480 and control cells was evaluated after treatment with 2 µM STLC or 100 nM BI6727. After treatment, cells were fixed and stained with the indicated antibodies. Scale bar, 5 µm. The kinetochore intensities of BUBR1 and MAD1 staining in the different treatment groups were quantified. The intensities were normalized to ACA. Values were calculated from at least 50 cells per treatment (means±s.d., $n$=3, for each concentration). *$P$<0.05, **$P$<0.01, ***$P$<0.001, Student's $t$-test, unpaired and two-tailed. **(c)** (left panel) Lysates of APC-ΔC-expressing SW480 and control cells treated with BI6727 were immunoblotted for MAD2, BUB3, p55-CDC20, Flag, and β-Actin. (right panel) Immunoprecipitation of CDC20 from asynchronous and BI6727 (100 nM)-treated SW480 and APC-ΔC-expressing SW480 cells. CDC20-interacting proteins were analyzed using western blot for MAD2, BUB3, BUBR1, and p55-CDC20.

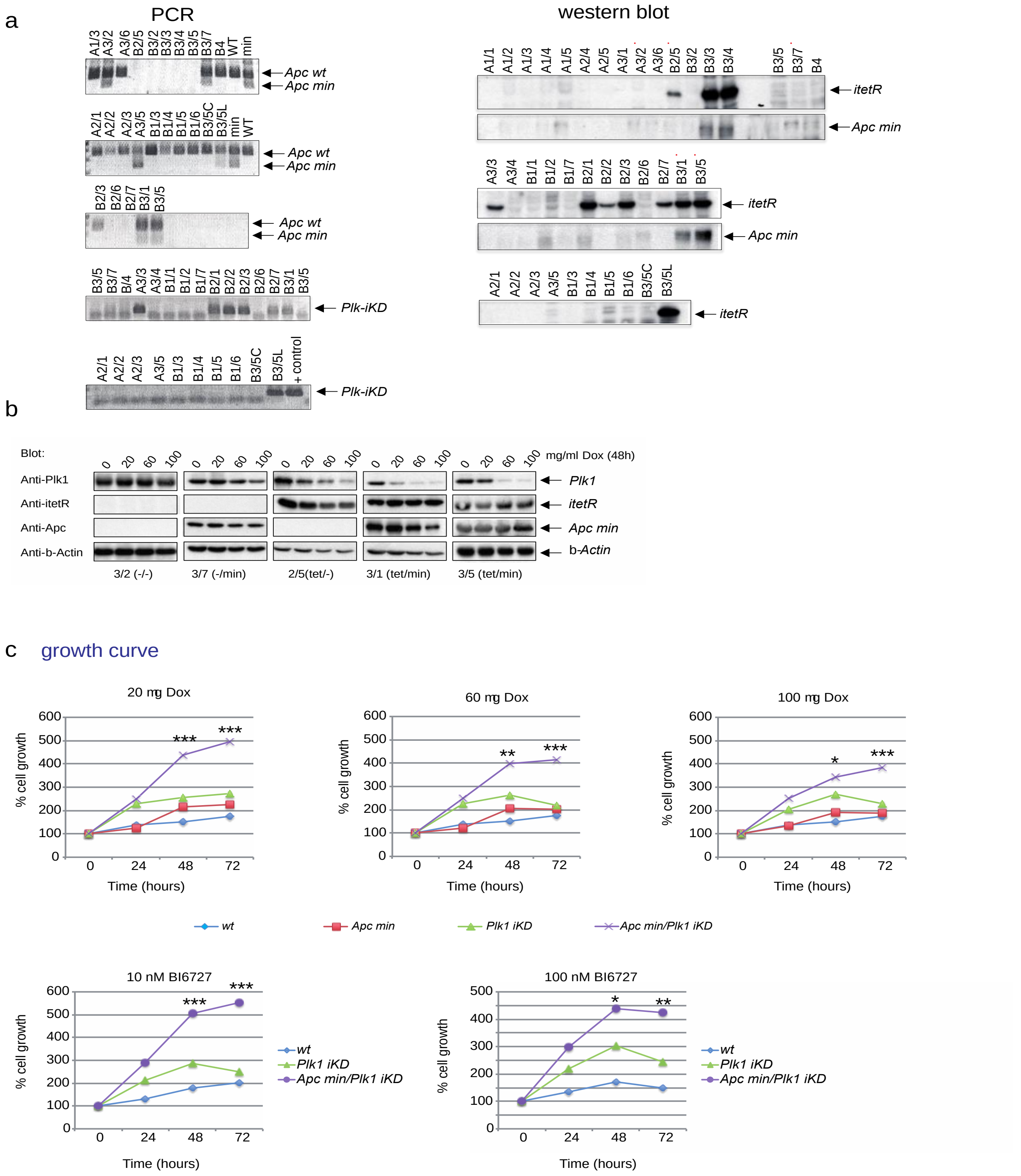


**Supplementary Figure 10**: **Correlation of PLK1 inhibition and the proliferative activity of mouse embryo fibroblasts (MEFs).**

(**a**) Analysis of MEF clones for *Apc* status and for the presence of the inducible *Plk1*-knockdown cassette (*Plk1 iKD*) by PCR. Mouse genomic DNA was isolated from MEFs derived from wild-type (wt), $Plk1^{iKD}$, $Apc^{Min/+}$ $Plk1^{iKD}$ and $Apc^{Min/+}$ mice and subsequently subjected to a PCR analysis (left) and Western Blot (right) to monitor for *Apc* (WT, min) and for *Plk1 iKD*. **(b)** Western blot analysis of *Plk1* expression in different MEF clones (wt, $Plk1^{iKD}$, $Apc^{Min/+}$ $Plk1^{iKD}$ and $Apc^{Min}$) at increasing Dox concentrations (20-100 µg/ml) is shown. **(c)** Proliferative activity of MEFs at different Dox (20-100 µg/ml) and BI6727 concentrations. (means±s.d., *n*=3, for each concentration). *$P$<0.05, **$P$<0.01, ***$P$<0.001, Student's *t*-test, unpaired and two-tailed.

Model:

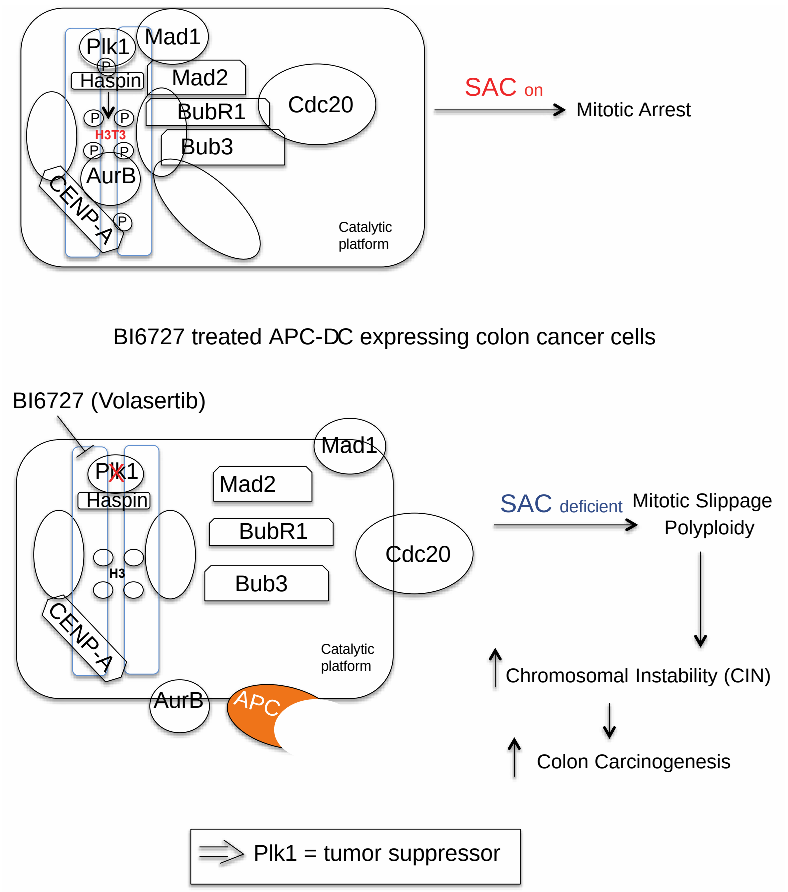


**Supplementary Figure 11: Schematic illustration of the combined effect of APC-ΔC-expression and PLK1 inhibition in HCT116 and SW480 cells.**

PLK1 inhibition in APC-ΔC-expressing cells lead to kinetochore mislocalization of Aurora B and to an impaired SAC activity due to a reduced recruitment of its components BUBR1 and MAD2 to kinetochores. This causes mitotic slippage and enhances the chromosomal instabilities in both cell lines.

| Days elapsed | Plk1 low | Plk1 high | Days elapsed | Plk1 low | Plk1 high |
|---|---|---|---|---|---|
| 926 | 0 | | 1095 | | 0 |
| 926 | 0 | | 2792 | | 0 |
| 740 | 1 | | 122 | | 1 |
| 1935 | 0 | | 182 | | 0 |
| 61 | 0 | | 926 | | 0 |
| 1849 | 1 | | 775 | | 0 |
| 718 | 0 | | 31 | | 0 |
| 371 | 0 | | 1024 | | 0 |
| 682 | 1 | | 1776 | | 0 |
| 335 | 0 | | 518 | | 0 |
| 441 | 0 | | 1518 | | 1 |
| 395 | 0 | | 3974 | | 0 |
| 1260 | 1 | | 3641 | | 0 |
| 338 | 1 | | 2587 | | 0 |
| 31 | 0 | | 962 | | 0 |
| 1004 | 0 | | 1915 | | 0 |
| 1162 | 1 | | 1829 | | 0 |
| 543 | 0 | | 388 | | 0 |
| 824 | 0 | | 1522 | | 0 |
| 500 | 0 | | 1127 | | 0 |
| 256 | 1 | | 383 | | 0 |
| 945 | 0 | | 1856 | | 0 |
| 403 | 1 | | 1883 | | 0 |
| 751 | 0 | | 792 | | 0 |
| 1247 | 0 | | 228 | | 1 |
| 435 | 0 | | 378 | | 0 |
| 1186 | 0 | | 542 | | 0 |
| 275 | 0 | | 669 | | 0 |
| 337 | 0 | | 1581 | | 0 |
| 457 | 0 | | 740 | | 1 |
| 457 | 0 | | 730 | | 0 |
| 1661 | 1 | | 59 | | 1 |
| 2683 | 0 | | 914 | | 0 |
| 580 | 0 | | 761 | | 0 |
| 617 | 0 | | 734 | | 0 |
| 0 | 0 | | 0 | | 0 |
| 685 | 0 | | 573 | | 0 |
| 2506 | 0 | | 650 | | 0 |
| 396 | 0 | | 791 | | 0 |
| 255 | 0 | | 518 | | 0 |
| 395 | 0 | | 609 | | 0 |
| 858 | 1 | | 4502 | | 0 |
| 2895 | 0 | | 1612 | | 0 |
| 385 | 0 | | 0 | | 0 |
| 427 | 1 | | 805 | | 0 |
| 1095 | 1 | | 426 | | 1 |
| 159 | 1 | | 578 | | 0 |
| 179 | 1 | | 834 | | 0 |
| 670 | 0 | | 3324 | | 0 |
| 1127 | 0 | | 31 | | 0 |
| 368 | 1 | | | | |
| 31 | 0 | | | | |

**Supplementary Table 1**: **Prism data files containing the survival analysis.**
